# Good Breeding

**Published:** 1896-06

**Authors:** 


### Good Breeding.

In good breeding, which differs, if at all, from high breeding only as it gracefully remembers the rights of others, rather than gracefully insists on its own rights, I discover no special connection with wealth or birth; but rather that it lies in human nature itself, and is due from all men toward all men.—*Thomas Carlyle.*

